# Development of 454 New Kompetitive Allele-Specific PCR (KASP) Markers for Temperate *japonica* Rice Varieties

**DOI:** 10.3390/plants9111531

**Published:** 2020-11-10

**Authors:** Kyeong-Seong Cheon, Young-Min Jeong, Hyoja Oh, Jun Oh, Do-Yu Kang, Nyunhee Kim, Eungyeong Lee, Jeongho Baek, Song Lim Kim, Inchan Choi, In Sun Yoon, Kyung-Hwan Kim, Yong Jae Won, Young-il Cho, Jung-Heon Han, Hyeonso Ji

**Affiliations:** 1 Department of Agricultural Biotechnology, National Institute of Agricultural Sciences, Rural Development Administration (RDA), Jeonju 54874, Korea; kscheon16@korea.kr (K.-S.C.); hja-oh@hanmail.net (H.O.); ospreys@nate.com (J.O.); 636597@naver.com (D.-Y.K.); knh702@korea.kr (N.K.); wowlek44@korea.kr (E.L.); firstleon@korea.kr (J.B.); greenksl5405@korea.kr (S.L.K.); inchchoi@korea.kr (I.C.); isyoon@korea.kr (I.S.Y.); biopiakim@korea.kr (K.-H.K.); jungheon1@rda.go.kr (J.-H.H.); 2 Seed Industry Promotion Center, Foundation of Agri. Tech. Commercialization & Transfer (FACT), Gimje 54324, Korea; ymjeong@efact.or.kr; 3 Cheorwon Branch, National Institute of Crop Science, Rural Development Administration (RDA), Cheorwon 24010, Korea; yjwon@korea.kr; 4 Seed Business Team, Department of Seed Services, Foundation of Agri. Tech. Commercialization & Transfer (FACT), Iksan 54667, Korea; breedy01@efact.or.kr

**Keywords:** temperate *japonica* rice, SNP, high-throughput genotyping, KASP

## Abstract

Temperate *japonica* rice varieties exhibit wide variation in the phenotypes of several important agronomic traits, including disease resistance, pre-harvest sprouting resistance, plant architecture, and grain quality, indicating the presence of genes contributing to favorable agronomic traits. However, gene mapping and molecular breeding has been hampered as a result of the low genetic diversity among cultivars and scarcity of polymorphic DNA markers. Single nucleotide polymorphism (SNP)-based kompetitive allele-specific PCR (KASP) markers allow high-throughput genotyping for marker-assisted selection and quantitative trait loci (QTL) mapping within closely related populations. Previously, we identified 740,566 SNPs and developed 771 KASP markers for Korean temperate *japonica* rice varieties. However, additional markers were needed to provide sufficient genome coverage to support breeding programs. In this study, the 740,566 SNPs were categorized according to their predicted impacts on gene function. The high-impact, moderate-impact, modifier, and low-impact groups contained 703 (0.1%), 20,179 (2.7%), 699,866 (94.5%), and 19,818 (2.7%) SNPs, respectively. A subset of 357 SNPs from the high-impact group was selected for initial KASP marker development, resulting in 283 polymorphic KASP markers. After incorporation of the 283 markers with the 771 existing markers in a physical map, additional markers were developed to fill genomic regions with large gaps between markers, and 171 polymorphic KASP markers were successfully developed from 284 SNPs. Overall, a set of 1225 KASP markers was produced. The markers were evenly distributed across the rice genome, with average marker density of 3.3 KASP markers per Mbp. The 1225 KASP markers will facilitate QTL/gene mapping and marker-assisted selection in temperate *japonica* rice breeding programs.

## 1. Introduction

Cultivated rice (*Oryza sativa* L.) is one of the most important crop species worldwide, providing food for more than half the global population. Cultivated rice consists of two main subspecies, *indica* and *japonica*. Geographically, *indica* rice is adapted to and mostly grown in tropical regions, and constitutes the majority of global rice production [1]. By contrast, *japonica* rice is divided into temperate *japonica* and tropical *japonica* cultivars, and temperate *japonica* rice is intensively cultivated and consumed in temperate regions of Asia including Korea, Japan, and parts of China [2]. In general, lower genetic diversity is observed in *japonica* varieties than in *indica* varieties [3,4]. Several types of molecular markers with high levels of polymorphism between *indica* and *japonica* cultivars have been developed, but the polymorphism of those markers within the *japonica* group is lower than within the *indica* group [2,3,5]. However, despite this low level of marker diversity, phenotypic differences in agronomic traits are apparent among closely related *japonica* rice varieties [6,7]. Because many of the rice genotyping markers that are polymorphic between *indica* and *japonica* varieties or between *indica* varieties lack polymorphism among the temperate *japonica* rice varieties [2,3], the availability of molecular markers for the construction of genetic maps and quantitative trait loci (QTL) analysis is limited for temperate *japonica* rice varieties. Thus, the identification of genome sequence variants and the development of efficient high-throughput markers are required for genetic analysis and molecular breeding of temperate *japonica* rice varieties.

Next-generation sequencing (NGS) technologies have enabled the discovery of numerous sequence variants between closely related genomes, and it became possible to develop enough polymorphic markers for genotyping in populations derived from crosses between closely related varieties [8,9,10,11]. Single nucleotide polymorphisms (SNPs) can be identified rapidly using whole genome sequencing. These SNPs can be used to develop high-throughput genotyping systems, and core SNP arrays have been developed for Japanese *japonica* rice [12,13] and *indica* rice [14]. The benefits of SNP array platforms include a range of multiplex levels providing rapid high-density genome scans, robust allele calling with high call rates, and cost-effectiveness per data point when genotyping large numbers of SNPs and samples [10,15]. However, the main disadvantages are that SNP arrays are non-flexible and, despite the low cost per data point, the overall cost for genotyping single samples remains high, making SNP arrays currently inaccessible for most breeding programs [15,16]. To compensate for the disadvantages of SNP arrays in breeding programs, several high-throughput and flexible SNP genotyping technologies have been developed. Of these, PCR-based fluorescently labeled SNP assays, such as TaqMan and kompetitive allele-specific PCR (KASP), are particularly useful as markers can be assessed individually, and results can be obtained using real-time PCR machines or fluorescent plate readers [10,16]. KASP assays are more cost-effective for genotyping than TaqMan systems, and were developed as an alternative to TaqMan with the objective of reducing cost and improving genotyping efficiency [10]. The KASP system is a single-step genotyping technology that uses pre-identified co-dominant alleles for both SNP and InDel variants [16], and has a scalability that makes it suitable for a wide range of experimental designs with extensively different target loci and sample numbers [16,17].

In our previous study, 740,566 SNPs distributed throughout the rice genome were identified from analysis of genome re-sequencing data of 13 Korean temperate *japonica* rice varieties [18]. Of the 740,566 SNPs, 1014 SNPs were chosen for KASP marker development by selection of SNP sites with polymorphism information content (PIC) values > 0.4 per 200 kbp. Of these, 506 SNP sites were used for KASP marker design, resulting in the successful development of 400 polymorphic KASP markers [18]. Subsequently, another 504 of the 1014 SNPs were used for additional KASP marker design, producing 371 KASP markers that were polymorphic among the tested varieties [19]. In total, 771 polymorphic KASP markers were developed for Korean temperate *japonica* rice varieties. These 771 KASP markers were successfully used for genetic map construction and QTL analysis of disease resistance and pre-harvest sprouting resistance in Korean temperate *japonica* rice varieties. For example, genetic maps were constructed using 205, 158, and 175 KASP markers with three F_2_ populations derived from crosses between Junam and Nampyeong, between Saenuri and Nampyeong, and between Junam and Samgwang, respectively. QTL analysis of bakanae disease resistance resulted in detection of three major QTLs on chromosomes 1, 6, and 9 [19,20]. A genetic map utilizing 239 KASP markers was constructed with 160 recombinant inbred lines (RILs) derived from a cross between two Korean temperate *japonica* varieties, Odae and Unbong40, and major QTLs for pre-harvest sprouting resistance were successfully detected on chromosomes 3, 4, and 11 [21]. The KASP markers were also used for genetic background analysis in Korean temperate *japonica* rice breeding. Marker-assisted backcrossing was used to facilitate the rapid development of near isogenic lines (NILs) to overcome the shortcomings of the parental Korean *japonica* rice variety Unkwang, which is susceptible to rice stripe virus (RSV). Unkwang was crossed to RSV-resistant variety Haedamssal as the donor of the RSV resistance gene, and the genetic backgrounds of BC_2_F_1_ and BC_2_F_2_ plants were analyzed with KASP markers to enable selection of a NIL with 96.2% recovery of the recurrent parent genome [22]. In a separate program to breed rice with reduced stale flavors after storage, 406 KASP markers were used for genetic background analysis of Jeonju624, a NIL of Korean temperate *japonica* rice variety Saenuri with a null allele of lipoxygenase-3 introduced from donor line HR27873-AC12, and revealed 95.8% recovery of the recurrent parent genome [23].

Despite these recent advances, several large gaps between markers remained, and additional KASP markers for temperate *japonica* rice were needed to improve gene mapping resolution. The aim of this study was to produce additional KASP markers, with a focus on SNPs with predicted high-impact effects on gene function and SNPs in large gap regions between existing markers. In total, 454 new KASP markers were developed that, when combined with the 771 extant markers, resulted in a set of 1225 polymorphic KASP markers available for Korean temperate *japonica* rice varieties. These markers will improve mapping QTLs/genes and breeding with temperate *japonica* rice varieties.

## 2. Results

### 2.1. Prediction of Effects of SNPs on Gene Function

In our previous study, 740,566 SNPs were identified from genome sequence data of 13 Korean temperate *japonica* rice varieties [18]. In this study, the effects of these SNPs on gene function were predicted using the SnpEff program [24]. The impacts of the SNPs were categorized into four groups: high, moderate, modifier, and low, with the groups containing 703 (0.1%), 20,179 (2.7%), 699,866 (94.5%), and 19,818 (2.7%) SNPs, respectively (Table 1). Stop-gained SNPs were the most abundant SNP type in the high-impact group (382 SNPs), and frameshifts were the least abundant (11 SNPs) (Supplementary Table S1). All the moderate-impact SNPs were non-synonymous SNPs. In the modifier group, SNPs in upstream gene regions were most abundant (514,460 SNPs), and SNPs in non-coding transcribed exon regions were least abundant (1129 SNPs). Synonymous SNPs were the most abundant SNP type in the low-impact group (16,127 SNPs), and SNPs that maintained a stop codon were the least abundant (19 SNPs).

### 2.2. Development of KASP Markers

Previously, we developed 771 KASP markers for high-throughput genotyping of Korean temperate *japonica* rice varieties [18,19]. The aim of this study was to develop additional KASP markers based on high-impact SNPs. The high-impact SNP group contained 703 SNPs. SNPs were selected for KASP marker development on the basis of their discrete predicted gene functions. After exclusion of SNPs in genes described as “hypothetical protein” and “conserved hypothetical protein”, 357 high-impact SNPs were selected for further development. These were denoted with the prefix “KGH” (Supplementary Table S2). KASP assays using these 357 high-impact SNPs were successful for all except one SNP, for which amplification failed in all 15 Korean *japonica* varieties tested. In the remaining 356 KASP assays, 73 showed monomorphism, and 283 KASP assays (79.3%) were polymorphic in the tested varieties (Table 2 and Supplementary Table S3). Subsequently, after integration of the 283 polymorphic KGH KASP markers with the 771 previously developed KASP markers, 284 additional SNPs were randomly selected from large gap regions between markers and primers designed for KASP assays (denoted “KJS”) (Supplementary Table S2). Amplification failed for nine of the KJS KASP markers in the Korean *japonica* varieties tested. In the remaining 275 KJS KASP assays, 104 markers were monomorphic, and 171 markers (60.2%) were polymorphic in the tested varieties (Table 2 and Supplementary Table S3).

**Figure 1:**
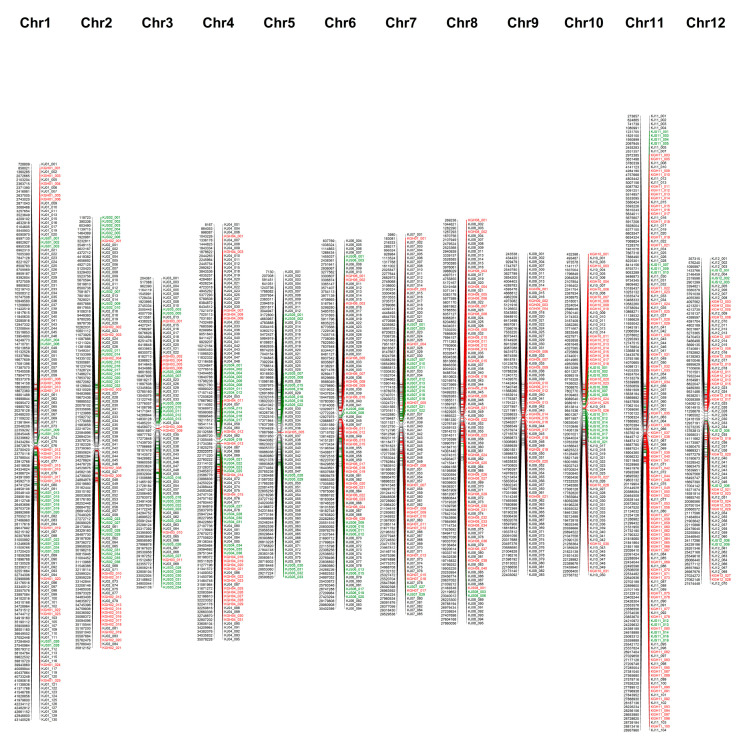
Physical map of 1225 KASP markers. Left and right sides of each chromosome indicate positions (bp) and names of markers, respectively. Black, red, and green text indicate KJ, KGH, and KJS KASP markers, respectively.

In total, 454 polymorphic KASP markers were developed from 641 SNPs (Figure 1). Of the 454 polymorphic KASP markers, 62.3% were high-impact SNP markers (frameshift, splice_acceptor, splice_donor, start_lost, stop_gained, stop_lost), 1.5% were moderate-impact SNP markers (non-synonymous), 35.0% were modifier SNP markers (5_prime_UTR, 3_prime_UTR, downstream_gene, upstream_gene, intergenic_region, intron_variant, and non_coding_transcript_exon), and 1.1% were low-impact SNP markers (synonymous and splice_region) (Table 3). Of the 1225 polymorphic KASP markers, 23.2% were for SNPs with predicted high-impact effects (frameshift, splice_acceptor, splice_donor, start_lost, stop_gained, stop_lost), 2.8% were for SNPs with moderate-impact effects (non-synonymous), 72.3% were for SNPs with modifier effects (5_prime_UTR, 3_prime_UTR, downstream_gene, upstream_gene, intergenic_region, intron_variant, non_coding_transcript_exon), and 1.7% were for SNPs with low-impact effects (synonymous, splice_region, and 5_prime_UTR_premature_start_codon_gain) (Table 3).

**Figure 2:**
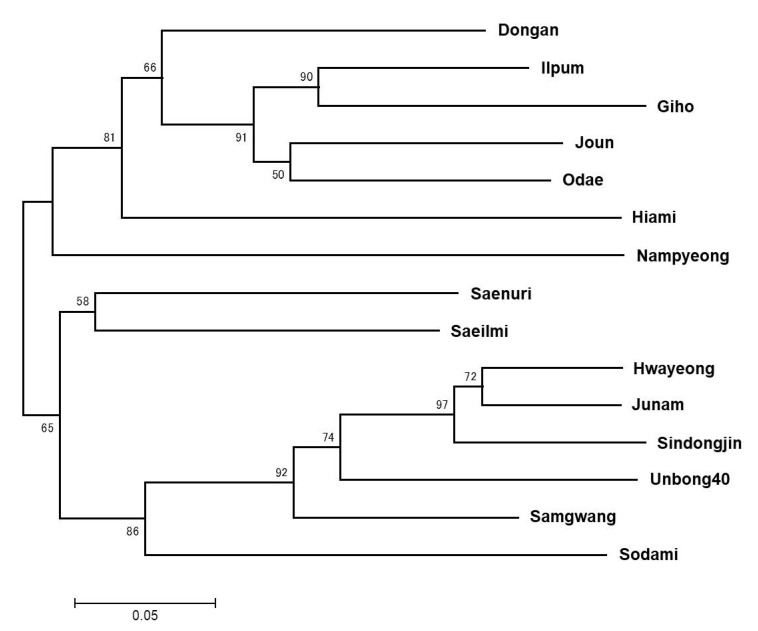
Phylogenetic analysis of 15 Korean temperate *japonica* rice varieties using genotyping data from 265 KASP markers. The phylogenetic tree was constructed using the neighbor-joining method. Numbers at the nodes indicate the percentage obtained with 1000 bootstraps.

A phylogenetic tree was generated for 15 *japonica* rice varieties tested with 265 KGH KASP markers (Figure 2). The 15 Korean *japonica* varieties were classified into three groups. Dongan, Ilpum, Giho, Joun, Odae, Hiami, and Nampyeong were included in the first group, Saenuri and Saeilmi were included in the second group, and Hwayeong, Junam, Sindongjin, Unbong40, Samgwang, and Sodami were included in the third group.

### 2.3. Integration of 1225 KASP Markers

**Figure 3:**
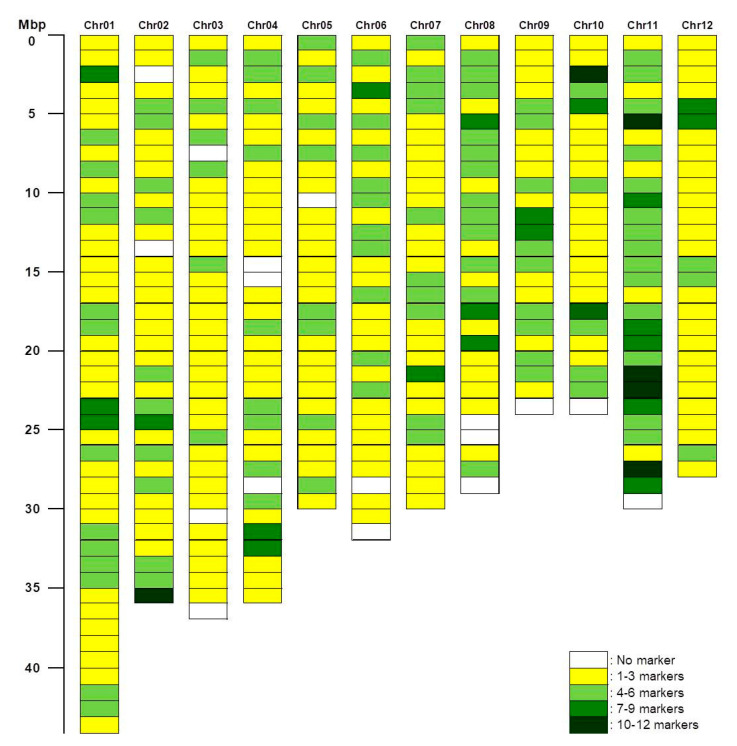
Distribution of 1225 polymorphic KASP markers developed with Korean temperate *japonica* rice varieties. Columns represent chromosomes, and rows represent physical position (1 Mbp intervals). Cell colors indicate the numbers of polymorphic KASP markers per Mbp (0–12).

In total, a set of 1225 polymorphic KASP markers comprising 771 previously developed KASP markers (“KJ”) and 454 KASP markers from this study was successfully developed (Table 4, Figure 1). The distribution of the 1225 polymorphic KASP markers across the rice genome was assessed. Overall, KASP markers were distributed relatively evenly, with an average density of 3.3 markers/Mbp across all the chromosomes (Supplementary Table S4). Particularly comprehensive coverage was observed for chromosomes 1, 7, and 12. However, large gaps of 1 Mbp remained on the other nine chromosomes, and 2 Mbp gaps were present on chromosomes 4 and 8 (Figure 3).

**Figure 4:**
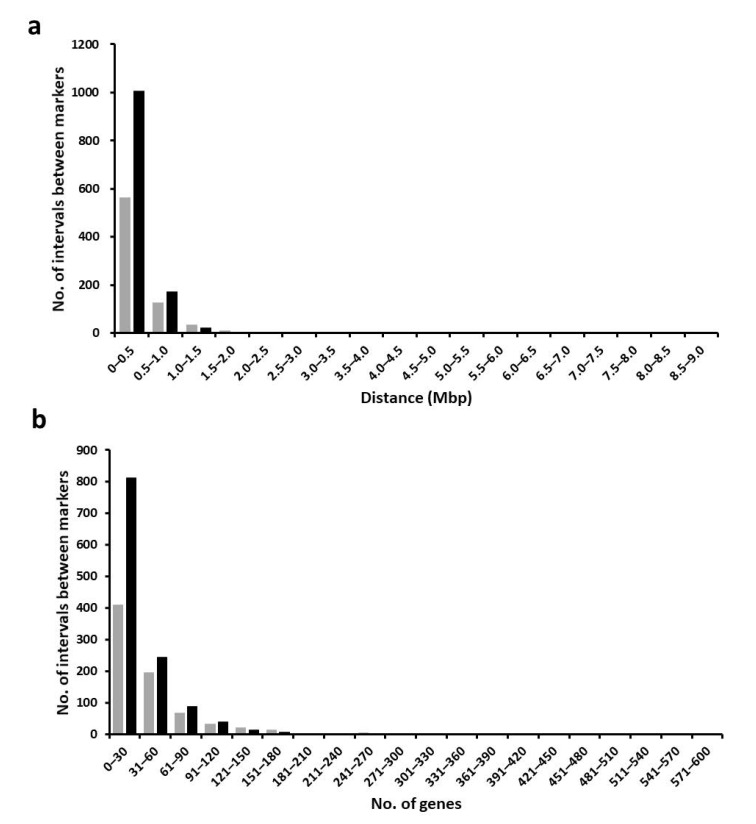
Histogram of distances and number of genes between markers. (**a**) Histogram of distances between markers. (**b**) Histogram of number of genes between markers. Grey bars indicate 771 KASP markers while black bars indicate 1225 KASP markers.

By increasing the numbers of developed KASP markers from 771 to 1225, the maximal distance between markers was reduced from 8.1 to 3.2 Mbp. In the histogram of distances between markers, the number of intervals between markers in 0–0.1 Mbp distance was increased while that over 0.1 Mbp distance was decreased in the physical map of 1225 KASP markers compared with that of 771 KASP markers (Figure 4a). Also, the maximal number of genes between markers was reduced from 593 to 415. In the histogram of number of genes between markers, the number of intervals between markers in 0–120 genes was increased while that over 120 genes was decreased in the physical map of 1225 KASP markers compared with that of 771 KASP markers (Figure 4b).

To assess the utility of the full 1225 KASP marker set for individual *japonica* rice crosses, the numbers of markers that were polymorphic in each cross combination were counted in crosses between 12 *japonica* rice varieties used in previous studies [18,19] and the present study (Table 5, Supplementary Table S3). With the exception of crosses with Unbong40, all cross combinations had more than 300 usable polymorphic markers. Unbong40 was analyzed with 825 KASP markers (371 previous and 454 new markers) (Table 4). In total, 300–399, 400–499, 500–599, and 600–694 polymorphic markers were usable for genotype analysis of 4, 12, 28, and 11 cross combinations, respectively. The largest and smallest numbers of usable polymorphic markers were detected in the Junam and Nampyeong cross (694 markers, 56.7%), and in the Hwayeong and Samgwang cross (310 markers, 25.3%), respectively.

## 3. Discussion

High-throughput genotyping markers enable genotyping to be conducted quickly and at a large scale, improving efficiency and facilitating molecular crop breeding programs. SNP-based markers are particularly useful for high-throughput genotyping of crops. Closely related crop cultivars with limited genetic diversity, such as temperate *japonica* rice, have highly homologous genomes but nevertheless retain a widespread distribution of SNPs across the genome. KASP analysis is a SNP genotyping platform that uses PCR and fluorescence detection and can support flexible, efficient, and cost-effective SNP genotyping [16]. In this study, KASP markers were developed for temperate *japonica* rice varieties. Temperate *japonica* rice varieties exhibit wide phenotypic variation for several important agronomic traits, including disease resistance, pre-harvest sprouting resistance, plant architecture, and grain quality, indicating the presence of agronomically favorable gene pools within the *japonica* germplasm. However, only a limited number of polymorphic traditional DNA markers have been identified, hampering gene mapping and molecular breeding of temperate *japonica* rice. The 1225 KASP marker set, which comprises 454 markers developed in this study and 771 markers developed previously, offers comprehensive genome coverage and will prove useful in breeding programs for temperate *japonica* rice varieties. Furthermore, we detected 20,179 non-synonymous SNPs with predicted moderate impact on gene function which have probability of being related with phenotype variation in important traits. These SNPs can be utilized for development of more KASP markers.

KASP assays for genotyping analysis have been developed for a range of plant species. In legumes, Saxena et al. [25] developed 1616 KASP markers and used them to screen 24 pigeonpea (*Cajanus cajan*) genotypes representing the parents of 14 mapping populations. Moreover, conversion of the SNPs to cost-effective and high-throughput KASP markers generated successful assays for 2005 SNPs in chickpea (*C. arietinum*). Screening of 70 genotypes, including 58 diverse chickpea accessions and 12 BC_3_F_2_ lines, showed that 1341 of the KASP markers were polymorphic [26]. In rice, 2144 candidate SNPs were identified that were polymorphic between *O. glaberrima* and *O. sativa*, 2015 of which were converted into KASP markers [27]. Of these 2015 KASP markers [27], 1890 KASP markers were found to be applicable to *indica* rice, and an additional 39 novel KASP markers were developed using nine *indica* rice varieties [17]. Filtering of several rice SNP datasets using eight criteria (*indica-indica* variation, high polymorphism levels, presence in functional genes, key gene targeting sites, cloned genic regions, important trait association, and gap filling) identified 596 SNPs, 467 of which were converted to KASP SNP markers [28].

In this study, KASP markers were developed for 641 SNP sites from 13 Korean temperate *japonica* rice genomes. Of these, 357 loci (KGH) were selected due to their predicted high-impact effects on gene function, and 284 loci (KJS) were selected at random to fill larger gaps between markers. Of these, 283 and 171 markers were polymorphic, respectively, in the tested *japonica* rice varieties. These markers were combined with 771 KASP markers that we developed previously, resulting in a set of 1225 polymorphic KASP markers for high-throughput SNP genotyping in temperate *japonica* rice (Table 4 and Figure 3). Of the 1225 polymorphic KASP markers, 25.3–56.7% were polymorphic in individual crosses between 12 Korean temperate *japonica* rice varieties (Table 5). The polymorphic markers in each individual cross were sufficient for mapping QTLs/genes, and polymorphism percentages were higher than those in other reports. In pairwise comparisons with nine *indica* rice varieties [17], 345–520 (18–28%) of the 1890 KASP markers were polymorphic. In interspecific crosses between CG14 (*O. glaberrim*) and WAB56-104 (*japonica* rice), and between TOG5681 (*O. glaberrima*) and IR64 (*indica* rice), 745 (37.0%) and 751 (37.3%) of the 2015 KASP markers were polymorphic, respectively [27].

In our previous studies, genetic maps were constructed for Korean temperate *japonica* rice varieties using subsets of the earlier 771 KASP markers. Genetic maps were constructed using 205, 158, and 175 KASP markers, with 188 F_2_ progenies derived from a cross between Junam and Nampyeong [18], between Saenuri and Nampyeong [19], and between Junam and Samgwang [20], respectively. A genetic map was also successfully constructed using 239 KASP markers with 160 RILs derived from a cross between Odae and Unbong40 [21]. However, additional SNP markers were needed in QTL target regions and in regions with large gaps between KASP markers, and several Cleaved Amplified Polymorphic Sequence (CAPS) markers were used to supplement the KASP-derived genetic maps [20,21]. The additional 454 KASP markers developed in this study will facilitate QTL/gene mapping and reduce the need for further CAPS markers.

Korean temperate *japonica* rice varieties experience low genetic diversity. This is likely due to high selection pressure for favorable agronomical traits and repeated use of elite lines with proven high yield or disease resistance in breeding programs, resulting in a limited genetic background and minimal *japonica* rice gene pool [7,29]. Analysis of the genetic relationships between 15 Korean temperate *japonica* rice varieties using 265 KASP markers divided cultivars into three groups, one of which contained six varieties: Hwayeong, Junam, Sindongjin, Unbong40, Sodami, and Samgwang (Figure 2). These grouping patterns were consistent with those observed in other studies of *japonica* rice varieties using SSR and KASP markers [18,30]. Several Korean temperate *japonica* rice varieties, including Junam, Sindongjin, Unbong40, Sodami, Samgwang, Saenuri, and Saeilmi, were derived from the Hwayeong cultivar in breeding programs [31]. However, despite this, the Saenuri and Saeilmi varieties were in a separate group to the Hwayeong group (Figure 2). The Saenuri and Saeilmi varieties were also derived from the Milyang 95 cultivar [30,31], suggesting that these two cultivars may have received a higher proportion of their genome from Milyang 95 than from Hwayeong. This phylogenetic analysis is consistent with other reports, and indicates that a small number of elite parents may have formed the basis of Korean temperate *japonica* rice breeding programs, resulting in high levels of genetic similarity among Korean temperate *japonica* varieties. The 265 KASP markers used for this phylogenetic analysis, and the full set of 1225 KASP markers, will facilitate the analysis of genetic relationships between even closely related Korean temperate *japonica* rice varieties.

## 4. Materials and Methods

### 4.1. Plant Materials and Genomic DNA Extraction

Fifteen Korean *japonica* rice varieties, Samgwang, Saenuri, Odae, Nampyeong, Junam, Ilpum, Hwayeong, Hiami, Joun, Giho, Sindongjin, Saeilmi, Sodami, Unbong40, and Dongan, were grown in a greenhouse of the National Institute of Agricultural Sciences (NIAS) of the Rural Development Administration (RDA, Jeonju, Korea) with maximum/minimum temperatures of 32/22 °C and light/dark lengths of 14/10 h. Genomic DNA for genotyping analysis was extracted from the leaves of ten seedlings per rice variety using a DNeasy Plant mini Kit (QIAGEN, Hilden, Germany).

### 4.2. SNP Impact Prediction and Selection of SNPs for KASP Marker Design

Genome sequence data from 13 Korean temperate *japonica* rice varieties were analyzed previously, and 740,566 SNPs were identified. The impacts of the 740,566 SNPs on gene function were predicted using the SnpEff program version 4.3f (http://snpeff.sourceforge.net/) [24]. The Nipponbare IRGSP-1.0 sequence and annotation (http://rapdb.dna.affrc.go.jp/download/irgsp1.html) from the Rice Annotation Project Database (RAP-DB, http://rapdb.dna.affrc.go.jp/) was used as the reference genome [32]. The SnpEff program can be used to annotate and classify polymorphisms according to their predicted effects on annotated genes, such as synonymous or non-synonymous changes, start codon gain or loss, and stop codon gain or loss. SnpEff can also classify polymorphisms according to their genomic locations, such as within intronic, 5′UTR, 3′UTR, upstream, downstream, or intergenic regions. The 740,566 SNPs were grouped into four categories according to their predicted impacts (high, moderate, modifier, and low) using the SnpEff program. SNPs with high-impact effects were selected, and those in genes with discrete predicted molecular function descriptions other than “hypothetical protein” or “conserved hypothetical protein” were used for KASP marker design (denoted “KGH”). After integrating the KGH markers and 771 previously developed KASP markers, additional SNPs were selected in large gap regions between markers and were used for KASP marker design (denoted “KJS”). The primers were designed based on flanking sequences of SNPs and manufactured by LGC Genomics (London, UK).

### 4.3. KASP Marker Assay 

The designed KASP markers were tested with 15 Korean temperate *japonica* rice varieties. KASP amplifications and allelic discriminations were performed using a Nexar system (LGC Douglas Scientific, Alexandria, USA) in the Seed Industry Promotion Center (Gimje, Korea) of the Foundation of Agricultural Technology Commercialization and Transfer. KASP assays were performed using 0.8 μL of 2× Master Mix and 0.02 μL of 72× KASP assay mix (LGC Genomics, London, UK) with 5 ng genomic DNA in a final reaction volume of 1.6 μL in a 384-well Array Tape. Reactions were performed in duplicate, and non-template controls were included in each run. KASP amplification was performed using the following thermal cycling profile: 15 min at 94 °C, followed by a touchdown phase of 10 cycles at 94 °C for 20 s and 61–55 °C (dropping 0.6 °C per cycle) or 68–62 °C (dropping 0.6 °C per cycle) for 60 s, followed by 26 cycles at 94 °C for 20 s and 55 °C or 62 °C for 60 s (first PCR stage). Next, recycling was performed with three cycles of 94 °C for 20 s and 57 °C for 60 s (second PCR stage). Recycling was performed two times, and the fluorescence measurement was taken for KASP genotyping after PCR amplification. Genotypes of each sample were called using Intellics software (LGC Douglas Scientific, Alexandria, USA) and then verified by manual inspection. Markers showing clear allelic discrimination were regarded as polymorphic, and those showing poor allelic discrimination were regarded as monomorphic.

### 4.4. Construction of a Physical Map and Phylogenetic Tree

SNP position data of polymorphic KASP markers in 15 Korean *japonica* rice varieties were used to construct a physical map using MapChart version 2.32 software [33]. Phylogenetic analysis of the 15 Korean *japonica* varieties was performed with genotyping data from 265 KASP markers by the Neighbor-joining method (1000 bootstraps), using POPTREE2 software [34].

## 5. Conclusions

A set of 1225 polymorphic KASP markers based on SNPs was developed for temperate *japonica* rice varieties. The set of KASP markers comprised 771 previously developed markers and 454 markers developed in this study. The 1225 KASP markers were evenly distributed across the rice genome, with an average marker density of 3.3 KASP markers per Mbp. The KASP markers developed here will be useful for mapping studies, will facilitate the identification of genes for favorable agronomical traits in temperate *japonica* rice varieties, and will improve rice molecular breeding programs.

## Figures and Tables

**Table 1:** Classification of single nucleotide polymorphisms (SNPs) by predicted effects on gene function.

	Impact of SNP Effects ^a^				Sum ^b^
	High	Moderate	Modifier	Low	
1	54 (7.7%)	1839 (9.1%)	41,820 (6.0%)	1805 (9.1%)	45,518 (6.1%)
2	43 (6.1%)	981 (4.9%)	19,906 (2.8%)	982 (5.0%)	21,912 (3.0%)
3	18 (2.6%)	450 (2.2%)	19,924 (2.8%)	460 (2.3%)	20,852 (2.8%)
4	45 (6.4%)	1566 (7.8%)	37,565 (5.4%)	1498 (7.6%)	40,674 (5.5%)
5	11 (1.6%)	438 (2.2%)	15,094 (2.2%)	474 (2.4%)	16,017 (2.2%)
6	53 (7.5%)	1570 (7.8%)	87,427 (12.5%)	1465 (7.4%)	90,515 (12.2%)
7	32 (4.6%)	1118 (5.5%)	36,872 (5.3%)	1181 (6.0%)	39,203 (5.3%)
8	96 (13.7%)	2209 (10.9%)	101,463 (14.5%)	2252 (11.4%)	106,020 (14.3%)
9	41 (5.8%)	1009 (5.0%)	36,503 (5.2%)	1033 (5.2%)	38,586 (5.2%)
10	65 (9.2%)	1781 (8.8%)	68,970 (9.9%)	1624 (8.2%)	72,440 (9.8%)
11	186 (26.5%)	5459 (27.1%)	152,754 (21.8%)	5158 (26.0%)	163,557 (22.1%)
12	59 (8.4%)	1759 (8.7%)	81,568 (11.7%)	1886 (9.5%)	85,272 (11.5%)
Sum ^c^	703 (0.1%)	20,179 (2.7%)	699,866 (94.5%)	19,818 (2.7%)	740,566 (100%)

^a^ The high-impact group includes frameshift, splice_acceptor, splice_donor, start_lost, stop_ gained, and stop_lost SNPs. The moderate-impact group includes non-synonymous SNPs (nsSNP). The modifier group includes 5_prime_untranslated_region (UTR), 3_prime_UTR, downstream_gene, upstream_gene, intergenic, intron, and non-coding_transcript_exon SNPs. The low-impact group includes 5_prime _UTR_ premature_start_codon_gain, synonymous SNP, splice_region, and stop_retained SNPs. Even though 5_prime _UTR_ premature_start_codon_gain SNPs were categorized into the low-impact group by the SnpEff program, they may be considered to have moderate or high impacts because they may cause changes in peptide sequences. Number (percentage of chromosome). ^b^ Number (percentage of chromosome). ^c^ Number (percentage).

**Table 2:** Summary of validation of designed kompetitive allele-specific PCR (KASP) markers with 15 Korean *japonica* rice varieties.

Name	Type	No.	%
KGH	Assay	357	
	Polymorphism	283	79.3
	Monomorphism	73	20.4
	No amplification	1	0.3
KJS	Assay	284	
	Polymorphism	171	60.2
	Monomorphism	104	36.6
	No amplification	9	3.2
Total	Assay	641	
	Polymorphism	454	70.8
	Monomorphism	177	27.6
	No amplification	10	1.6

**Table 3:** Classification of KASP markers by their SNP effects.

Impact of SNP Effect	SNP Effect	Marker Set	
		454 KASPs	1225 KASPs
HIGH	Frameshift	7	7
	Splice_acceptor	25	25
	Splice_donor	29	29
	Start_lost	12	12
	Stop_gained	147	147
	Stop_lost	63	64
	Sum	283	284
MODERATE	Missense	7	34
MODIFIER	5_prime_UTR	1	9
	3_prime_UTR	4	25
	Downstream_gene	4	31
	Upstream_gene	131	708
	Intergenic_region	16	97
	Intron_variant	1	12
	Non_coding_transcript_exon	2	4
	Sum	159	886
LOW	5_prime_UTR_premature_start_codon_gain ^a^	0	3
	Synonymous	4	16
	Splice_region	1	2
	Sum	5	21

^a^ Even though 5_prime _UTR_ premature_start_codon_gain SNPs were categorized into the low-impact group by the SnpEff program, they may be considered to have moderate or high impacts because they may cause changes in peptide sequences.

**Table 4:** Summary of KASP assays with Korean temperate *japonica* rice varieties.

	Name	Assay (*n*)	Polymorphism (*n*, %)	Monomorphism (*n*, %)	No Amplification (*n*, %)	Reference
1st	KJ	506	400 (79.1)	89 (17.6)	17 (3.4)	[18]
2nd	KJ	504	371 (73.6)	126 (25.0)	7 (1.4)	[19]
3rd	KGH, KJS	641	454 (70.8)	177 (27.6)	10 (1.6)	this study
Total		1651	1225 (74.2)	392 (23.7)	35 (2.1)	

**Table 5:** The number and percentage of polymorphic KASP markers for genotype analysis of cross combinations between 12 Korean temperate *japonica* rice varieties. These varieties were used in common in the present and previous studies.

	DA	HA	HY	IP	JU	JN	GH	NP	OD	SG	SN	UB
**DA**	-											
**HA**	523 (42.7)	-										
**HY**	483 (39.4)	544 (44.4)	-									
**IP**	579 (47.3)	526 (42.9)	600 (49.0)	-								
**JU**	546 (44.6)	487 (39.8)	688 (56.2)	440 (35.9)	-							
**JN**	561 (45.8)	527 (43.0)	345 (28.2)	466 (38.0)	613 (50.0)	-						
**GH**	554 (45.2)	537 (43.8)	521 (42.5)	388 (31.7)	444 (36.2)	529 (43.2)	-					
**NP**	382 (31.2)	587 (47.9)	543 (44.3)	662 (54.0)	509 (41.6)	694 (56.7)	653 (53.3)	-				
**OD**	557 (45.5)	505 (41.2)	661 (54.0)	400 (32.7)	410 (33.5)	575 (46.9)	530 (43.3)	659 (53.8)	-			
**SG**	454 (37.1)	577 (47.1)	310 (25.3)	609 (49.7)	600 (49.0)	449 (36.7)	544 (44.4)	528 (43.1)	598 (48.8)	-		
**SN**	437 (35.7)	578 (47.2)	428 (34.9)	590 (48.2)	625 (51.0)	555 (45.3)	582 (47.5)	441 (36.0)	574 (46.9)	510 (41.6)	-	
**UB ^a^**	316 (38.3)	321 (38.9)	265 (32.1)	376 (45.5)	370 (44.8)	289 (35.0)	340 (41.2)	378 (45.8)	324 (39.2)	273 (33.1)	322 (39.0)	-

DA, Dongan; HA, Hiami; HY, Hwayeong; IP, Ilpum; JU, Joun; JN, Junam; GH, Giho; NP, Nampyeong, OD, Odae; SG, Samgwang; SN, Saenuri; UB, Unbong40. Number and percentage of polymorphic KASP markers from the 1225 markers used are shown. ^a^ Crosses involving Unbong40 used 825 polymorphic KASP markers and percentages were calculated accordingly.

## Supplementary Materials

The following are available online at https://www.mdpi.com/2223-7747/9/11/1531/s1, Table S1: Classification of SNPs by effects on gene function, Table S2: Sequence and position of 641 KASP primer sets, Table S3: Genotypic analysis of 15 Korean *japonica* varieties using KASP markers, Table S4: Density of 1225 KASP markers within each chromosome.
