# Supplementary material for: Development of 454 New Kompetitive Allele-Specific PCR (KASP) Markers for Temperate japonica Rice Varieties
Source: Plants (Basel). 2020 Nov 10;9(11):1531. doi: 10.3390/plants9111531 (PMC7698039; doi:10.3390/plants9111531)
Supplement: Supplementary file 1 [file plants-09-01531-s001.zip › Table_S1_SNP_calssification.docx]

Table S1. Classification of SNPs by effects on gene function.

| Impacts of SNP effects | SNP effects | Chr01 | Chr02 | Chr03 | Chr04 | Chr05 | Chr06 | Chr07 | Chr08 | Chr09 | Chr10 | Chr11 | Chr12 | Total |
| --- | --- | --- | --- | --- | --- | --- | --- | --- | --- | --- | --- | --- | --- | --- |
| HIGH | Frameshift | 1 | 1 | 0 | 0 | 0 | 0 | 0 | 0 | 0 | 2 | 7 | 0 | 11 |
|  | Splice_acceptor | 7 | 1 | 1 | 4 | 1 | 7 | 4 | 8 | 3 | 6 | 11 | 8 | 61 |
|  | Splice_donor | 4 | 8 | 0 | 7 | 0 | 4 | 0 | 11 | 9 | 6 | 12 | 4 | 65 |
|  | Start_lost | 5 | 1 | 3 | 4 | 5 | 2 | 1 | 6 | 1 | 5 | 10 | 2 | 45 |
|  | Stop_gained | 27 | 23 | 7 | 21 | 4 | 27 | 22 | 58 | 19 | 36 | 105 | 33 | 382 |
|  | Stop_lost | 10 | 9 | 7 | 9 | 1 | 13 | 5 | 13 | 9 | 10 | 41 | 12 | 139 |
| MODERATE | Non-synonymous | 1,839 | 981 | 450 | 1,566 | 438 | 1,570 | 1,118 | 2,209 | 1,009 | 1,781 | 5,459 | 1,759 | 20,179 |
| MODIFIER | 5_prime_UTR | 612 | 327 | 175 | 596 | 118 | 882 | 330 | 997 | 390 | 638 | 1,832 | 835 | 7,732 |
|  | 3_prime_UTR | 1,207 | 626 | 482 | 1,163 | 359 | 1,682 | 809 | 2,185 | 893 | 1,465 | 4,059 | 1,542 | 16,472 |
|  | Downstream_gene | 2,077 | 887 | 946 | 2,142 | 855 | 6,719 | 2,252 | 7,853 | 2,348 | 4,335 | 8,791 | 5,899 | 45,104 |
|  | Upstream_gene | 33,645 | 16,191 | 15,757 | 27,448 | 12,200 | 61,226 | 27,519 | 73,091 | 26,317 | 51,334 | 111,796 | 57,936 | 514,460 |
|  | Intergenic_region | 3,796 | 1,711 | 2,127 | 5,375 | 1,408 | 15,321 | 5,191 | 15,724 | 5,833 | 9,995 | 23,311 | 14,581 | 104,373 |
|  | Intron | 436 | 152 | 391 | 803 | 130 | 1,464 | 715 | 1,415 | 664 | 1,078 | 2,718 | 630 | 10,596 |
|  | Non_coding_transcript_exon | 47 | 12 | 46 | 38 | 24 | 133 | 56 | 198 | 58 | 125 | 247 | 145 | 1,129 |
|  | 5_prime_UTR_premature_start_codon_gain | 111 | 42 | 33 | 97 | 21 | 150 | 65 | 200 | 67 | 114 | 355 | 167 | 1,422 |
|  | Synonymous | 1,509 | 815 | 358 | 1,211 | 393 | 1,169 | 944 | 1,772 | 838 | 1,329 | 4,304 | 1,485 | 16,127 |
|  | Splice_region | 183 | 123 | 69 | 190 | 59 | 144 | 171 | 278 | 128 | 178 | 494 | 233 | 2,250 |
|  | Stop_retained | 2 | 2 | 0 | 0 | 1 | 2 | 1 | 2 | 0 | 3 | 5 | 1 | 19 |
| Total |  | 45,518 | 21,912 | 20,852 | 40,674 | 16,017 | 90,515 | 39,203 | 106,020 | 38,586 | 72,440 | 163,557 | 85,272 | 740,566 |
